# Ligation-induced DNA self-assembly

**DOI:** 10.1093/nar/gkaf570

**Published:** 2025-07-04

**Authors:** Qishu Zhang, Zhengyang Sun, Yaduo Wang, Lei Xue, Jianqiu Zhao, Yue Shen, Bryan Wei, Wen Wang

**Affiliations:** School of Life Sciences, Tsinghua University, Beijing 100084, China; Center for Synthetic and Systems Biology, Tsinghua University, Beijing 100084, China; School of Life Sciences, Tsinghua University, Beijing 100084, China; Center for Synthetic and Systems Biology, Tsinghua University, Beijing 100084, China; School of Life Sciences, Tsinghua University, Beijing 100084, China; Center for Synthetic and Systems Biology, Tsinghua University, Beijing 100084, China; BGI Research, Beijing 102601, China; BGI Research, Shenzhen 518083, China; BGI Research, Changzhou 213299, China; School of Life Sciences, Tsinghua University, Beijing 100084, China; Center for Synthetic and Systems Biology, Tsinghua University, Beijing 100084, China; BGI Research, Beijing 102601, China; BGI Research, Shenzhen 518083, China; BGI Research, Changzhou 213299, China; School of Life Sciences, Tsinghua University, Beijing 100084, China; Center for Synthetic and Systems Biology, Tsinghua University, Beijing 100084, China; BGI Research, Beijing 102601, China; BGI Research, Shenzhen 518083, China; BGI Research, Changzhou 213299, China

## Abstract

Ligation is a common treatment for the resulting DNA nanostructures to gain extra stability. In this study, we aim to utilize ligation as an active process in self-assembly instead of post-assembly stabilization. Our investigation focuses on constructs with transiently paired segments. Transient base pairing fails to hold up the assembled complex, but ligation treatment turns the transient base pairing into permanent ones and thus induces the desired self-assembly. We apply the method to a number of assembly tasks, leading to the successful construction of discrete and extended DNA nanostructures. Furthermore, we apply the ligation-based method in the hierarchical assembly of preformed DNA nanostructure units into higher-order superstructures.

## Graphical Abstract

**Graphical Abstract:**
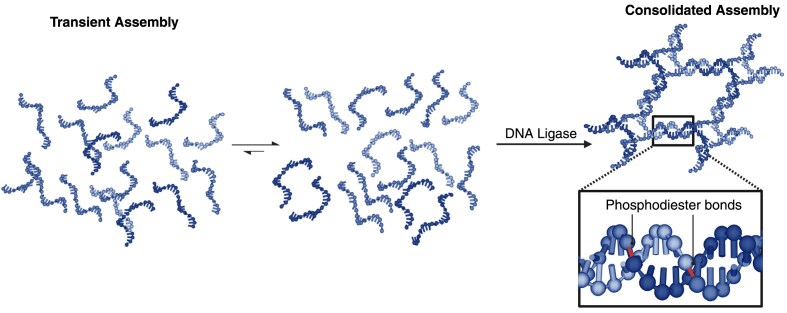

## Introduction

The simple rule of Watson–Crick base pairing has allowed for a massive collection of increasingly complex and organized constructs in DNA nanotechnology [1–5]. Since synthetic DNA molecules share the same chemical composition as their natural counterparts, the entire toolbox of DNA modifying enzymes from molecular biology can be seamlessly applied to DNA nanotechnology. Enzymatic treatments have already enabled a number of static and dynamic constructs. For example, polymerases [6–13], restriction enzymes [14, 15], and ligases [10, 16–21] are among the many enzymes that render structural and functional features to the resulting DNA nanostructures.

DNA ligase is one of the most widely investigated enzyme types for DNA nanostructures. Because most DNA nanostructures are composed of multiple short strands, nicks are ubiquitous for duplexes with segment-to-segment base pairing. When a certain nick between neighboring segments is sealed by ligase, the corresponding segments are merged into a longer one, which becomes less dissociation prone. As a consequence, ligation can provide stability benefits to the resulting constructs [17–19]. Besides the stabilizing role, ligation can also be used to trigger structural reconfiguration of DNA nanostructures [10, 19–21]. However, ligation was mostly just a post-assembly treatment to provide certain structural features to the assembled constructs. A direct assembly role of this enzymatic treatment is still underexplored.

**Figure 1.**
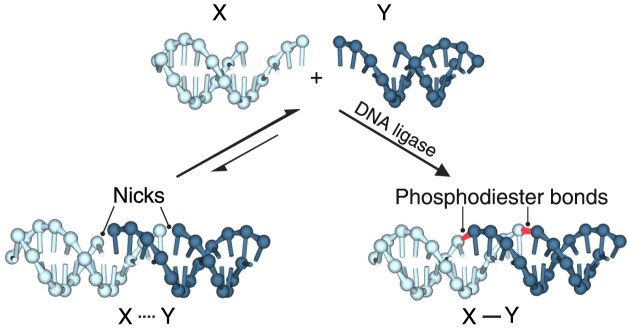
Schematics of the ligation-induced assembly method. The cohesion of a short sticky end is reversible, and the dissociation of the base pairing partners is the favored reaction direction. Upon ligation, the sticky end cohesion reaction becomes irreversible, resulting in a permanent assembly.

In this work, we introduce a ligation-induced assembly method (Fig. 1). Instead of serving as a post-assembly treatment for structural stabilization, ligation is directly involved in the self-assembly process, inducing the combination of short segments into longer ones and eventually forming the designated connecting network collectively. We focus on base pairing interactions normally regarded as transient. Specifically, the lengths of the key segments to be paired (e.g. sticky ends) are set to be no more than five nucleotides. In the absence of ligase, the pairing of short segments and corresponding connection network remains transient. In the presence of ligase, on the other hand, transient hybridizations are gradually consolidated with an emerging connection network and thus the desired construct realized.

Following such a scheme, we successfully produced many DNA nanostructures in our ligation-induced self-assembly. These constructs failed to self-assemble in typical hybridization-based assembly without ligase but became achievable with the ligation. A variety of discrete and extended structures composed of junction motifs are constructed. To further demonstrate the versatility of this ligation-based method, we applied the same strategy to hierarchically assemble preformed DNA nanostructure units into higher-order superstructures.

## Materials and methods

### Sequence design

The DNA strands were designed using Uniquimer [22] or Cadnano2 [23] software and purchased from Integrated DNA Technologies and Azenta Life Sciences. DNA sequences can be found in the Supplementary data.

### Ligation-induced assembly

T4 DNA ligase seals nicks in DNA by catalyzing the formation of a covalent phosphodiester bond between the 5′ phosphate group of one DNA strand and the 3′ hydroxyl group of another at the nick site. To enable this activity, 5′ phosphate modification was provided by T4 polynucleotide kinase (PNK). In this study, selected DNA strands, with a final concentration of 500 nM, were incubated with PNK (20 U per 20 μl) in a 1× T4 DNA ligase buffer containing 50 mM Tris–HCl (pH 7.5), 10 mM MgCl_2_, 10 mM dithiothreitol, and 1 mM adenosine triphosphate (ATP) at 37°C for 5 h to achieve 5′ phosphorylation, followed by incubation at 65°C for 10 min to inactivate the kinase. Reaction duration was systematically optimized through time-course experiments ranging from 30 min to 20 h and the 5-h incubation is consistent with the protocol from our earlier study [21].

For the assembly of four-arm discrete and extended structures, the phosphorylated DNA strands, with a final concentration of 100 nM, were annealed directly with the addition of T4 DNA ligase (400 U per 20 μl). Both the ligase-treated and control samples were incubated under identical experimental conditions at 30°C for 16 h in 1× T4 DNA ligase buffer, differing only in the presence or absence of T4 DNA ligase. Since T4 DNA ligase is stored in a buffer with 50% glycerol, an equivalent volume (1 μl) of 50% glycerol was added to the control groups in the initial experiments (Supplementary Figs S1 and S4) to match the glycerol content introduced by T4 DNA ligase-treated sample. Subsequent analyses showed that glycerol had no detectable impact on assembly outcomes. Therefore, glycerol was not supplemented in control groups for the rest of the study. Reaction conditions were systematically for protocol optimization: supplemental ATP (0.5–1.0 M, in addition to the buffer’s intrinsic 1 M ATP), T4 DNA ligase dosage (600–800 U per 20 μl reaction), Mg^2+^ concentration (20–100 mM), the inclusion or exclusion of 0.5 M betaine as a molecular crowding agent, incubation time (30 min to 20 h), and incubation temperature (20–35°C for lattices and 4°C for origami).

For hierarchical assemblies (5 × 5 lattices and snowflake-shaped origami), units were first annealed. In the case of the 5 × 5 lattices, the phosphorylated strands were mixed with the units in 0.5× TE buffer with 20 mM MgCl_2_ and annealed using a slow ramp program from 90°C to 25°C, over 17 h. For the snowflake-shaped origami, the phosphorylated strands were mixed with the units purified from 1% agarose gel in 0.5× TE buffer with 12.5 mM MgCl_2_ and annealed from 85°C to 25°C, over 2.5 h. At this stage, each DNA strand was at a concentration of 100 nM. Following these annealing steps, ligation-induced assembly of DNA motif proceeded under the same conditions as those used for four-arm discrete and extended structures.

### Gel electrophoresis and purification

Agarose gels were run in 0.5× TBE buffer with 10 mM MgCl_2_ in an ice water bath. The gels were detected by Amersham Typhoon scanner (GE Healthcare Bio-Sciences AB) equipped with 488-nm laser and Cy2 filter.

Target bands were excised under cyan light, crushed in Freeze’N Squeeze columns (Bio-Rad), and then centrifuged directly at 1000 rpm for 2 min at 4°C.

### Thermal stability assessment

Samples of ligation induced assemblies were heated at varying temperatures ranging from 40°C to 90°C for 4 h. After thermal treatment, the resulting products were immediately analyzed using 2% non-denaturing agarose gel electrophoresis. Experimental controls (maintained at 4°C) along with a 1-kb DNA size marker (Thermo Scientific) were included as comparative migration standards.

### Quantitative analysis of assembly yield and melting curves

Gel image analysis was performed using ImageJ (v1.49) to calculate the intensity-based assembly yield by quantifying the ratio of the target band signal to the total lane signal. Background subtraction (rolling ball radius: 50 pixels) was applied, followed by manual selection of the target band and entire lane regions. Integrated density values for both regions were measured, and the yield (%) was calculated as


\begin{eqnarray*}
{\mathrm{Yield = }}\frac{{\rm {IDV}_{target}}}{{\rm {IDV}_ {lane}}} \times 100\% .
\end{eqnarray*}

Survival rates were determined by normalizing the intensity of the target band to that of the control band (4°C reference) using agarose gel electrophoresis data. Melting curve profiles were generated and analyzed with OriginPro (version 2018).

### AFM imaging

The morphology of the DNA structures was characterized by atomic force microscopy (AFM) using a Multimode 8 system (Bruker) in liquid ScanAsyst mode. A 10 μl droplet of purified sample and a 50 μl droplet of 1× TAE buffer containing 20 mM MgCl_2_ were applied to a freshly cleaved mica surface. To enhance DNA–mica binding, a 10 μl droplet of 10 mM NiCl_2_ was added to the mica surface. Additional dilution of the sample was performed sometimes to achieve the desired sample density.

### TEM imaging

A 10 μl droplet of purified sample was applied to a carbon-coated grid (Electron Microscopy Sciences) that had undergone plasma treatment. The excess was wicked away after 2 min incubation and stained for 15 s with 10 μl of 2% aqueous uranyl formate. The stain buffer was then removed using filter paper and the grid was left to dry in open air. The stained sample was analyzed by Hitachi 7650B, operated at 80 kV.

## Results

**Figure 2.**
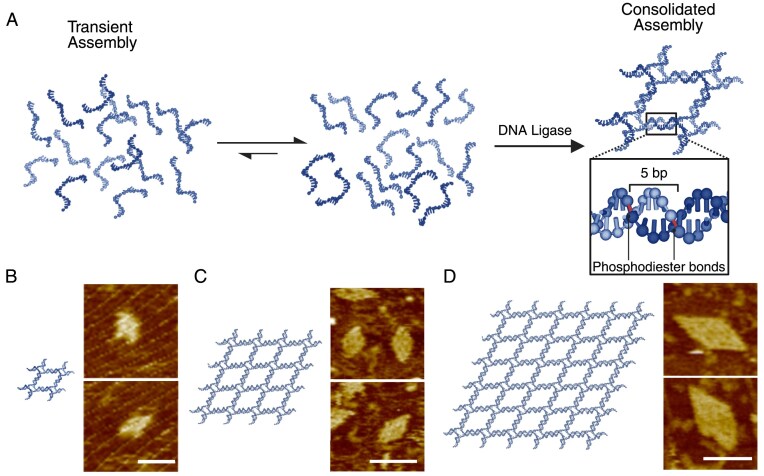
Ligation-induced assembly of discrete lattices composed of junction motifs. (**A**) Schematics of ligation-induced assembly of a 2 × 2 lattice. Transiently bound 5-nt sticky ends (see Supplementary data for kinetic simulation), which lead to a state of partial assembly, are consolidated by ligation, resulting in the designated assembly. Ligated sites are shown in the zoomed-in box. Schematic diagrams (left) and the corresponding AFM images (right) of discrete lattices: (**B**) 2 × 2 lattice; (**C**) 4 × 4 lattice; and (**D**) 6 × 6 lattice. Scale bars: 50 nm.

We first sought to design multiple four-arm junction motifs for the self-assembly of discrete DNA nanostructures [15]. A certain sticky end of each motif arm was designed as 5 nt, whose pairing with its designated partner is too transient for multiple motifs to assemble. The transient pairing can be consolidated by ligation treatment, enabling the proper inter-motif interactions. As an initial example, we investigated the self-assembly of a 2 (vertex) × 2 (vertex) lattice composed of four four-arm junction motifs with 5-nt sticky ends annealed with and without T4 DNA ligase (Fig. 2A). Agarose gel electrophoresis revealed a product band for the ligated sample, while no target band was present for the unligated samples assembled at temperatures ranging from 20°C to 35°C, demonstrating a direct assembly role of ligation treatment (Supplementary Figs S1C and S2). AFM imaging further confirmed the ligation-induced self-assembly (Fig. 2B and Supplementary Fig. S1B).

We then extended this approach to 4 × 4 and 6 × 6 lattices. Similar to the 2 × 2 lattice, these larger lattices failed to self-assemble under a typical annealing condition (isothermal annealing temperature range: 20–35°C, Supplementary Fig. S4), with no apparent target bands displayed in agarose gel electrophoresis for unligated samples (Supplementary Figs S2–S4). Upon the induction of T4 DNA ligase, the desired 4 × 4 and 6 × 6 lattices were successfully produced. The self-assembly was confirmed by agarose gel electrophoresis (Supplementary Figs S2–S4) and AFM imaging (Fig. 2C and D).

**Figure 3.**
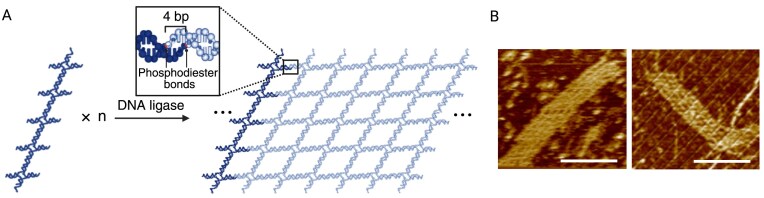
Ligation-induced assembly of extended lattice. (**A**) Schematic of extended lattice assembled from structural units composed of five four-arm junction motifs. Transiently paired 4-nt sticky ends are designed to be stabilized by ligation treatment. Ligated sites are shown in the zoomed-in box. (**B**) AFM images of the 1D extended lattice. Scale bars: 100 nm.

We next applied the ligation-based method for an extended construct (Fig. 3A). Within the repeating unit composed of five four-arm junction motifs (1 × 5 lattice), stably paired segments were adopted (10 or 11 base pairs). The inter-unit connection was designed as five pairs of 4-nt sticky ends (Fig. 3A and Supplementary Fig. S5). As indicated by agarose gel electrophoresis results, no target assembly was present for reaction without ligase and proper assembly was induced with ligase. AFM imaging confirmed the successful ligation-induced assembly, revealing ribbon-like structures extending up to 1 μm with an expected width and texture (Fig. 3B).

**Figure 4.**
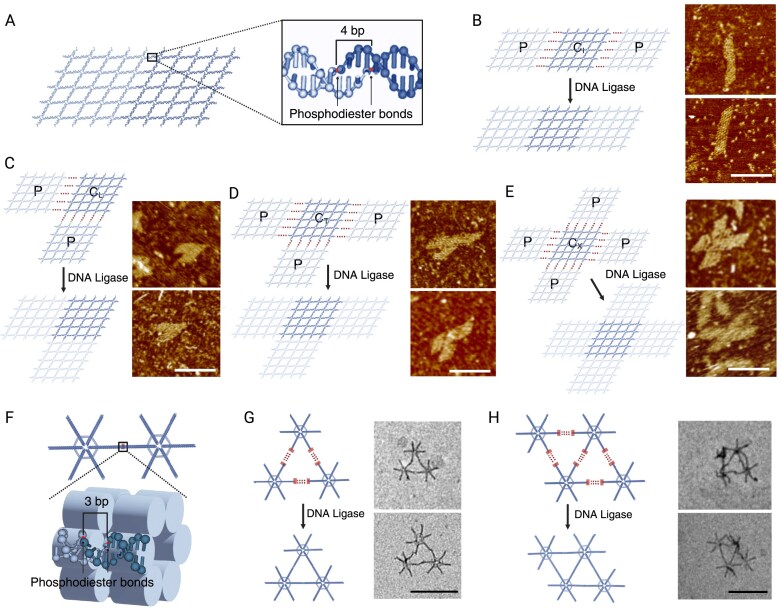
Ligation-induced hierarchical assembly of preformed structural units. (**A**) Schematic of the ligation-induced assembly of two preformed 5 × 5 lattices. Transiently paired 4-nt sticky ends are designed to be stabilized by ligation treatment. Ligated sites are shown in the zoomed-in box. Ligation-induced assembly of preformed 5 × 5 lattices into different shapes: (**B**) I-shaped trimers; (**C**) L-shaped trimers; (**D**) T-shaped tetramers; and (**E**) X-shaped pentamers. Schematics are shown on the left, with the corresponding AFM images on the right. Scale bars: 50 nm. (**F**) Schematic of the ligation-induced assembly of two preformed snowflake-shaped origami units. Transiently paired 3-nt sticky ends are designed to be stabilized by ligation treatment. Ligated sites are shown in the zoomed-in schematic. Ligation-induced assembly of origami units into different multimer patterns: (**G**) A triangular pattern from three origami units and (**H**) A diamond pattern from four origami units. Schematics are shown on the left, with the corresponding transmission electron microscopy (TEM) images on the right. Scale bars: 20 nm.

Furthermore, we implemented this strategy for the hierarchical assembly of multiple preformed DNA nanostructure units into higher-order superstructures. We adopted a preformed 5 × 5 lattice, composed of 25 four-arm junction motifs, as the structural unit for the assembly task. Each inter-unit sticky end was designed as 4 nt and five for each boundary edge (Fig. 4A and Supplementary Fig. S6). As already shown in the aforementioned example (Supplementary Fig. S5), five 4-nt sticky ends were not stable enough to hold up the assembled complex without ligation treatment. In the I-shaped trimer assembly, the center unit (C_I_) was designed with sticky ends (s_1_–s_5_) on its east and west boundaries. Two identical copies of the peripheral unit (P) were designed with complementary sticky ends (s_1_*–s_5_*) to match those on unit C_I_. Upon ligation, the sticky end cohesion joins unit C_I_ with two units P to form an I-shaped trimer (Fig. 4B and Supplementary Fig. S6).

Similarly, the combination of a unit C_L_ (center unit of L-shaped trimer) with sticky ends on its west and south boundaries with two copies of unit P led to the formation of an L-shaped trimer; a unit C_T_ (center unit of T-shaped trimer) with sticky ends on its east, west, and south boundaries with three copies of unit P led to the formation of a T-shaped tetramer; and C_X_ unit (center unit of X-shaped trimer) with sticky ends on its east, west, north, and south boundaries with four copies of unit P led to the formation of X-shaped pentamer (Supplementary Fig. S6). The successful self-assembly of all four shapes induced by ligation was confirmed by agarose gel electrophoresis (Supplementary Fig. S7, left panel) and AFM imaging (Fig. 4B–E). As a control group, typical sticky-end-based hierarchical assemblies of I-shaped, L-shaped, and T-shaped multimers were conducted, resulting in lower yields (Supplementary Fig. S7, right panel).

We also utilized this ligase-based strategy in hierarchical assembly of snowflake-shaped origami units [24] (Fig. 4F and Supplementary Fig. S8). As shown in Supplementary Fig. S9, two or three arms of a six-arm origami unit were designed with 3-nt sticky ends. To circumvent potential steric hindrance during the ligation reaction, four of the six helices in a specific bundled arm were appended with sticky ends. A specific arm-to-arm sticky end cohesion scheme corresponded to a designated origami multimer pattern. For example, a triangular trimer pattern resulted from the assembly of three origami units with sticky ends specified on two arms, and a diamond tetramer pattern resulted from the assembly of four origami units with sticky ends specified on two or three arms (Supplementary Fig. S9C). Upon ligation, the desired origami trimer and tetramer were generated in the ligation-induced hierarchical assembly. The origami multimer self-assembly induced by ligation was confirmed by agarose gel electrophoresis (Supplementary Figs S10 and S11) and TEM imaging (Fig. 4G and H). Notably, such an assembly was also achievable at 4°C (Supplementary Fig. S12), much lower than a typical annealing temperature.

## Discussion

In general, we developed a ligation-induced self-assembly method to build DNA nanostructures. Segments to be paired are carefully designed to be short enough that proper self-assembly is not achievable under typical hybridization conditions. When ligase was applied, the transiently paired segments covalently linked to the neighboring segments as longer and, therefore, more stably paired segments. Such a process applies to multiple short segments to be collectively paired, and the ligated segments result in a permanently connected network. Implementing this method, we were able to assemble complex nanostructures with transiently paired segments.

In general, our method enables DNA self-assembly based on sticky ends, which deems impossible in common hybridization-based methods. As such, a certain construct can be further miniaturized with shorter components but maintain the same number of structural features (e.g. vertex number and edge number). Besides short segments to be arranged globally, DNA nanostructures can be designed with short segments and the corresponding nicks located at specific sites. The carefully designed local short segments can then result in a reconfiguration upon ligation, which can make geometric specifications [25–27] more controllable and dynamic.

The enabling of self-assembly based on short sticky ends by ligation has already been shown in molecular cloning [28, 29], in which multiple strands or multiple fragments with short sticky ends get combined effectively. In the case of Golden Gate assembly, the length of sticky ends (i.e. 4 nt) for fragments to be assembled is determined by type IIS restriction enzyme [30] and impossible to be extended any longer. Since sticky end pairs of 4 bp would not hold the combined complexes, ligase becomes indispensable for the overall assembly process to take place in high efficiency. Conceptually, our method of DNA nanostructure self-assembly shares the same spirit with the molecular cloning methods to convert transient binding to permanent ones. Although sticky end length is adjustable for a certain DNA nanostructure, short sticky ends could become preferable especially in hierarchical assembly. Random aggregation and nonspecific inter-unit interactions can be contained with short sticky ends because of the transient nature for such pairing interactions [31]. Assembly yield benefit of our ligation-induced method with 4-nt sticky ends has already been shown in comparison against hierarchical assembly with 12-nt sticky ends (Supplementary Fig. S13 and Supplementary Table S1).

With an emerging stability induced gradually by individual ligation reactions, the pairing orthogonality and strength can be satisfied at the same time, resulting in a desired self-assembly. In terms of the assembly yield, stability, and integrity, the current strategy is in general comparable to traditional methods (those using stable long sticky ends for single-stranded DNA cohesion), as demonstrated by the experimental results (Supplementary Figs S14–S16). When parameters (e.g. sticky end and spacer lengths [32]) of the ligase induced hierarchical self-assembly are further optimized, such a system can bring in balanced pairing orthogonality and strength, and then it could become a useful method to build DNA nanostructures at higher level of complexity and order. Since the temperature for paired short sticky ends to hold the assembled complex without ligation is much lower than that required for the ligation reaction [33], the preferred temperature for ligation-induced assembly is hence determined by the permissible temperature of the ligation reaction. Practically, our method enables the DNA self-assembly at room temperature or lower. We believe many application opportunities in life and material sciences that require low temperatures will follow up.

In our system, enzyme actively contributes to the self-assembly process instead of post-assembly stabilization. The innovative utilization of ligase enables the process that is not realizable under typical hybridization conditions. Taking advantage of the large toolkit of DNA modifying enzymes, including many thermophilic and halophilic enzymes, DNA self-assembly assisted by enzymes can work in a wider range of temperature and ionic strength. With different modifications provided by diverse enzymes and proteins, rational design of reaction pathways with DNA rendered on the fly can offer energetic and kinetic benefits for a more controllable self-assembly.

## Supplementary Material

gkaf570_Supplemental_Files

## Data Availability

All data needed to evaluate the conclusions in the paper are present in the paper and/or Supplementary data.
