# Supplementary material for: Ligation-induced DNA self-assembly
Source: Nucleic Acids Res. 2025 Jul 4;53(12):gkaf570. doi: 10.1093/nar/gkaf570 (PMC12231581; doi:10.1093/nar/gkaf570)
Supplement: gkaf570_Supplemental_Files [file gkaf570_supplemental_files.zip › SI.pdf]

Ligation-induced DNA self-assembly

Qishu Zhang,[1, 2, †] Zhengyang Sun,[1, 2, †] Yaduo Wang,[1, 2] Lei Xue,[3, 4, 5] Jianqiu Zhao,[1, 2] Yue Shen,[3, 4, 5] Bryan Wei[1, 2, *] and Wen Wang[3, 4, 5, *]

[1] School of Life Sciences, Tsinghua University, Beijing 100084, China
[2] Center for Synthetic and Systems Biology, Tsinghua University, Beijing 100084, China
[3] BGI Research, Beijing 102601, China
[4] BGI Research, Shenzhen 518083, China
[5] BGI Research, Changzhou 213299, China
[†] Joint First Authors.
[*] To whom correspondence should be addressed. Email: wangwen4@genomics.cn (W.W.) and bw@tsinghua.edu.cn (B.W.).

This PDF file includes:

Supplementary Note S1
Supplementary Figures S1 to S16
Supplementary Table S1

Other Supplementary Information folders for this manuscript include the following:

Sequences

Table of Contents

Supplementary Note *3*

Note S1. Kinetic simulation of transient binding. 3

Supplementary Figures *4*

Figure S1. Assembly results of 4-arm (2 × 2) lattices with varying segment lengths, with and without DNA ligase treatment. 4

Figure S2. Comparison of T4 DNA ligation-induced assembly of 2 × 2, 4 × 4, and 6 × 6 lattices on a single gel. 5

Figure S3. DNA ligation-induced assembly of 4-arm 4 × 4 and 6 × 6 lattices. 6

Figure S4. Assembly of 4-arm lattice using T4 DNA ligase. 7

Figure S5. Ligation-induced assembly of the 5 × n infinite extension structure. 8

Figure S6. Hierarchical assembly of 5 × 5 lattices using ligation-induced method. 9

Figure S7. Gel electrophoresis results of the 5 × 5 lattices using ligation-induced method. 10

Figure S8. Cadnano diagram of snowflake origami unit. 11

Figure S9. Design and assembly of origami units with 3-nt sticky ends. 12

Figure S10. Gel electrophoresis and corresponding AFM images of ligation-induced assembly of snowflake trimer origami units. 13

Figure S11. Gel electrophoresis and corresponding AFM images of ligation-induced assembly of snowflake tetramer origami units. 14

Figure S12. Gel electrophoresis of snowflake origami tetramer structures at lower temperatures. 15

Figure S13. Assembly yields of design structures using ligation-induced method and traditional long sticky ends cohesion. 16

Figure S15. Comparison of structure integrity using ligation-induced method and traditional long sticky ends cohesion. 18

Figure S16. Thermal stability analysis of ligation-induced method and traditional long sticky ends cohesion. 19

Supplementary Table *20*

Table S1. Summary of design structures and assembly yields. 20

Supplementary Note

Note S1. Kinetic simulation of transient binding.

We employ kinetic simulation to elucidate the transient binding behavior of 5-nt sticky ends in the absence of ligase. Under standard reaction conditions (30°C, 10 mM Mg^{2+}, 100 nM substrate concentration), transiently bound 5-nt sticky ends exhibit a dwell time of approximately 1.7 seconds. The yield of a four-strand complex stabilized by two complementary 5-nt sticky ends is 0.14%, as indicated by NUPACK(1) analysis. These short-lived interactions initiate hybridization but dissociate rapidly.

Supplementary Figures

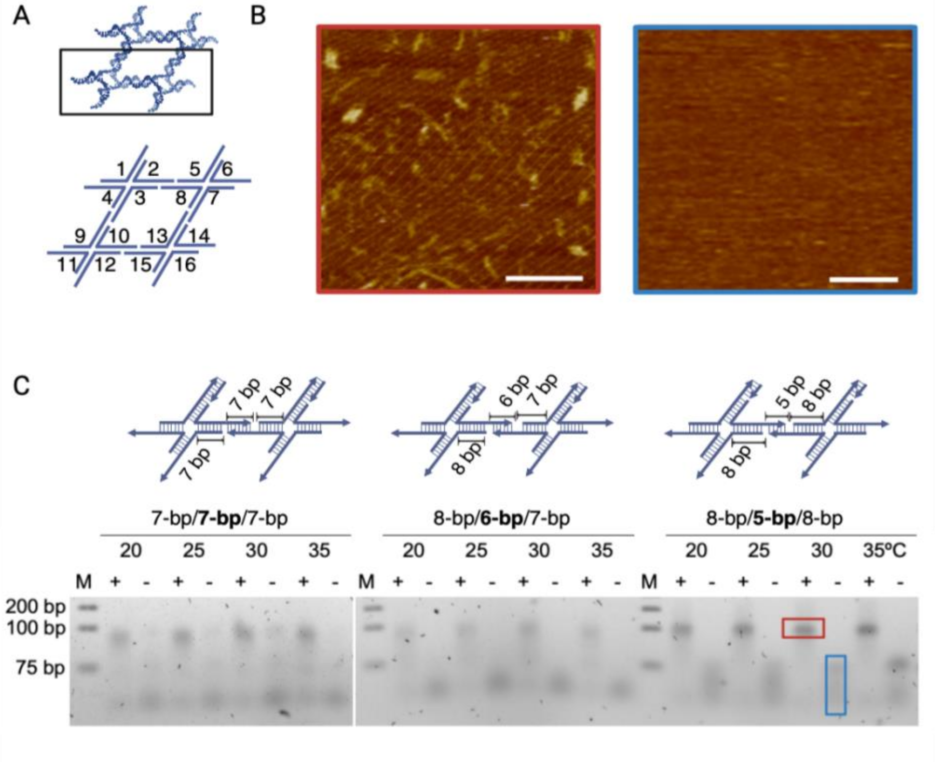

Figure S1. Assembly results of 4-arm (2 × 2) lattices with varying segment lengths, with and without DNA ligase treatment.

(**A**) 3D and 2D schematic representations of ligation-induced assembly of a 4-arm 2 × 2 structure. All strands have been phosphorylated at the 5′ ends. Different segment arrangements were designed to maintain a 21-bp edge length, specifically, 7-bp/**7-bp**/7-bp, 8-bp/**6-bp**/7-bp, and 8-bp/**5-bp**/8-bp configurations. These specific segment arrangements (in black box) correspond to the schematics in (**C**). (**B**) AFM images of the 4-arm 2 × 2 structures assembled with 5-nt sticky ends. Scale bars: 80 nm. The border colors correspond to the bands highlighted in (**C**). (**C**) Gel electrophoresis results of 4-arm 2 × 2 structures with different sticky edges lengths assembled with (+) and without (-) DNA ligase under identical experimental conditions. The specific segment arrangements depicted in (**A**) are reflected in the corresponding gel lanes, where differences in assembly efficiency can be observed. The 8-bp/**5-bp**/8-bp segmentation (5-nt sticky ends) follows a hierarchical assembly process, where stable 4-arm junctions formed first through the base pairing of the 8-bp segments, followed by the inter-junction assembly by the base pairing of two complementary 5-nt sticky ends. Such a process improved assembly yield compared to 7-bp/**7-bp**/7-bp (7-nt sticky ends) and 8-bp/**6-bp**/7-bp (6-nt sticky ends) segmentations.

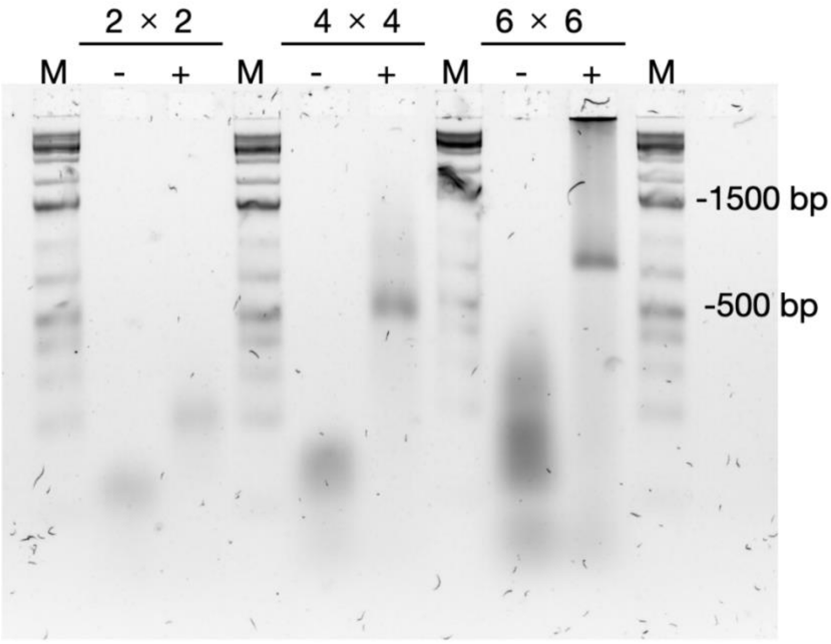

Figure S2. Comparison of T4 DNA ligation-induced assembly of 2 × 2, 4 × 4, and 6 × 6 lattices on a single gel.
Gel electrophoresis results illustrate the ligation-induced assembly of 2 × 2, 4 × 4, and 6 × 6 lattices. In each set, lanes marked with "+" indicate samples treated with T4 DNA ligase under standard assembly conditions (30°C incubation), demonstrating successful assembly. While the "-" lanes serve as controls incubated under identical conditions but without T4 DNA ligase, showing partial or no assembly.

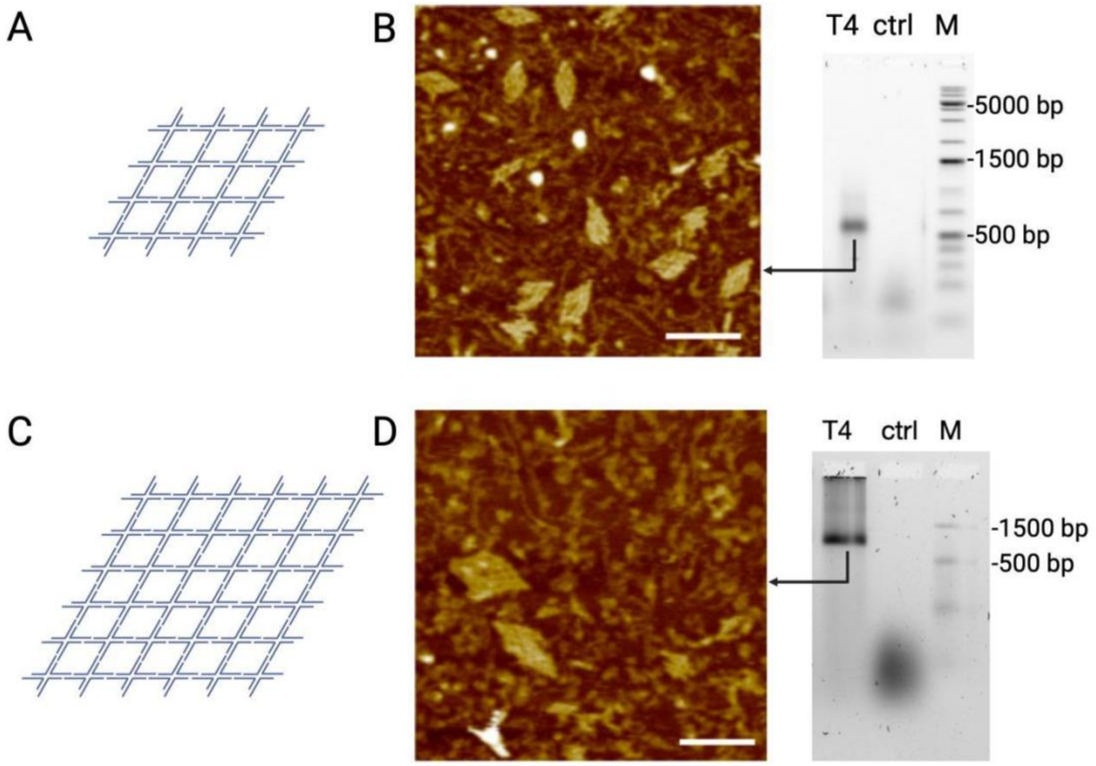

Figure S3. DNA ligation-induced assembly of 4-arm 4 × 4 and 6 × 6 lattices.
(**A**) 2D schematic of the DNA ligation-induced assembly of a 4-arm 4 × 4 lattices. All strands were phosphorylated at the 5' ends. (**B**) AFM image and corresponding gel electrophoresis result of the purified 4 × 4 lattice, with the arrow indicating the target band. (**C**) 2D schematic of the DNA ligation-induced assembly of a 4-arm 6 × 6 lattice. All strands were phosphorylated at the 5' ends. (**D**) AFM image and corresponding gel electrophoresis result of the purified 6 × 6 lattice, with the arrow indicating the target band. Scale bars: 80 nm.

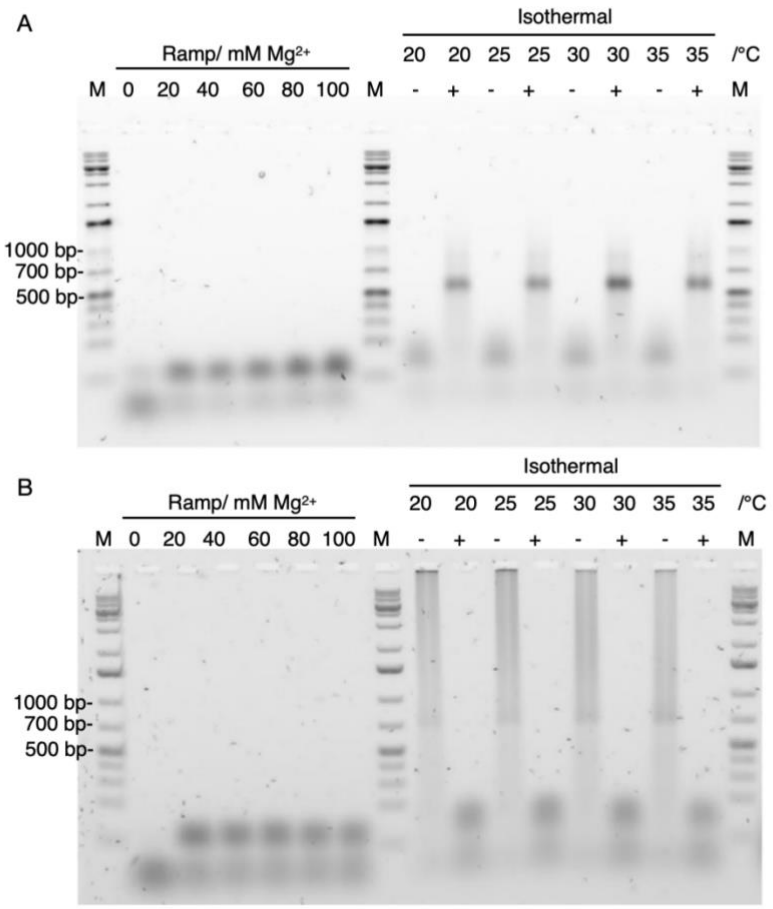

Figure S4. Assembly of 4-arm lattice using T4 DNA ligase.
(**A**) Gel electrophoresis of the 4 × 4 lattice assembly. Lane 1-6: Assembly without DNA ligase, following a traditional ramp annealing protocol (first ramp from 90ºC to 60ºC at a rate of 5 min/ºC, and second ramp from 60ºC to 25ºC at a rate of 25 min/ºC). Each lane corresponds to different magnesium concentrations of 0, 20, 40, 60, 80, and 100 mM, respectively. Lane 8, 10, 12, 14: Ligation-induced assembly with DNA ligase (+) incubated at temperatures of 20, 25, 30, and 35ºC, respectively. Lane 7, 9, 11, 13: Control experiments incubated at the same temperatures without DNA ligase (-). (**B**) Gel electrophoresis of the 6 × 6 lattice assembly. Lane 1-6: Assembly without DNA ligase, using the traditional ramp protocol with magnesium ion concentrations of 0, 20, 40, 60, 80, and 100 mM, respectively. Lane 7, 9, 11, 13: Ligation-induced assembly with DNA ligase (+) at 20, 25, 30, and 35ºC, respectively. Lane 8, 10, 12, 14: Control experiments at the same temperatures without DNA ligase (-).

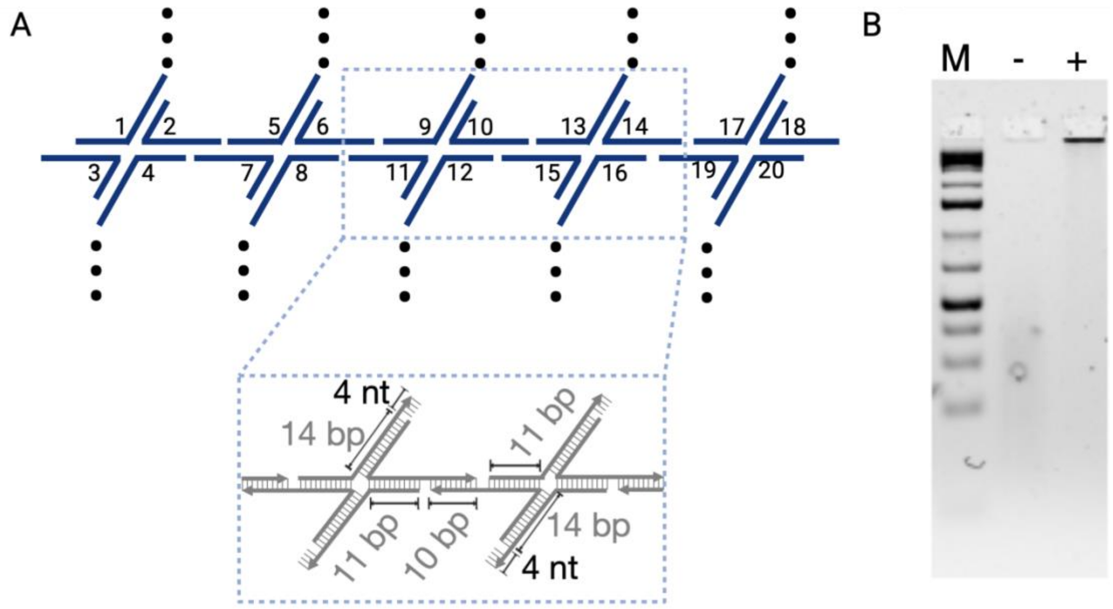

Figure S5. Ligation-induced assembly of the 5 × n infinite extension structure.
(**A**) 2D schematic representation of the 5 × n infinite extension structure with 4-nucleotide sticky ends between repeating units. Only the chosen strands (strands 2,3,6,7,10,11,14,15,18,19) were treated with PNK and phosphorylated. (**B**) Gel electrophoresis results showing the assembly of the 5 × n structure with and without T4 DNA ligase. The addition of ligase results in aggregation in well, indicating successful ligation and formation of the extended structure. Molecular weight marker (M) included for reference. "+" indicate samples treated with T4 DNA ligase, while "–" lanes serve as controls without ligase.

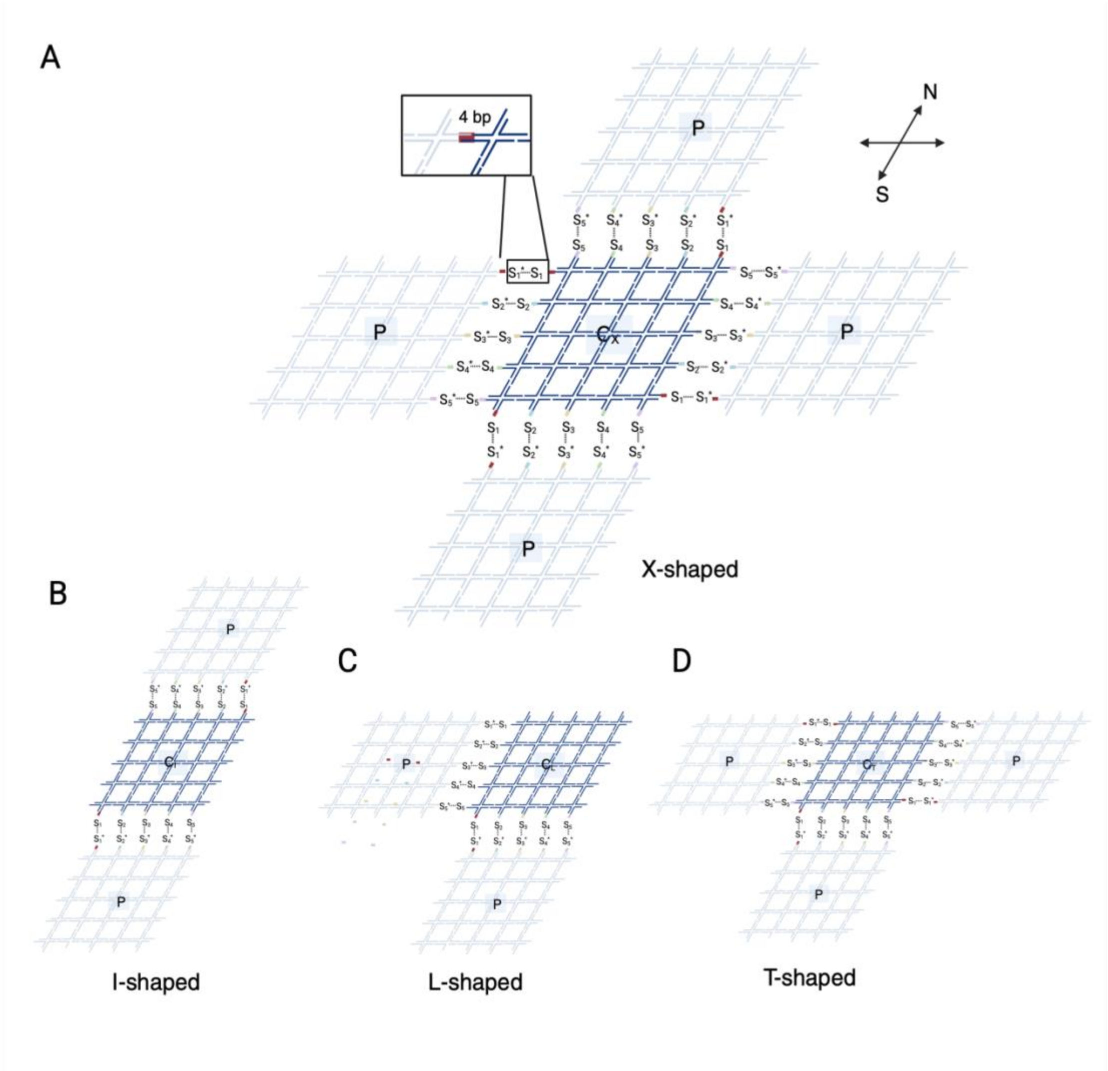

Figure S6. Hierarchical assembly of 5 × 5 lattices using ligation-induced method. 3D schematic of the 5 × 5 lattice pentamer assembly and 2D schematic illustrating the connectivity between units. The Peripheral unit (light blue) are identical, with the C_X unit (**A**) positioned at the center of the pentamer and designed with 4nt sticky ends (s_1-s_5) on all edges. Label 1-5 represent unique connection sites for different sequences. Other multimers shown in the study are derived from the C_X unit. The C_T design includes three ligatable edges, excluding the north edge SE of C_X (**D**), forming a tetramer with identical peripheral units upon ligation-induced assembly. Similarly, C_I includes the east and west SE sequences of C_X (**B**), while C_L includes the west and north SE sequences (**C**), forming respective trimers.

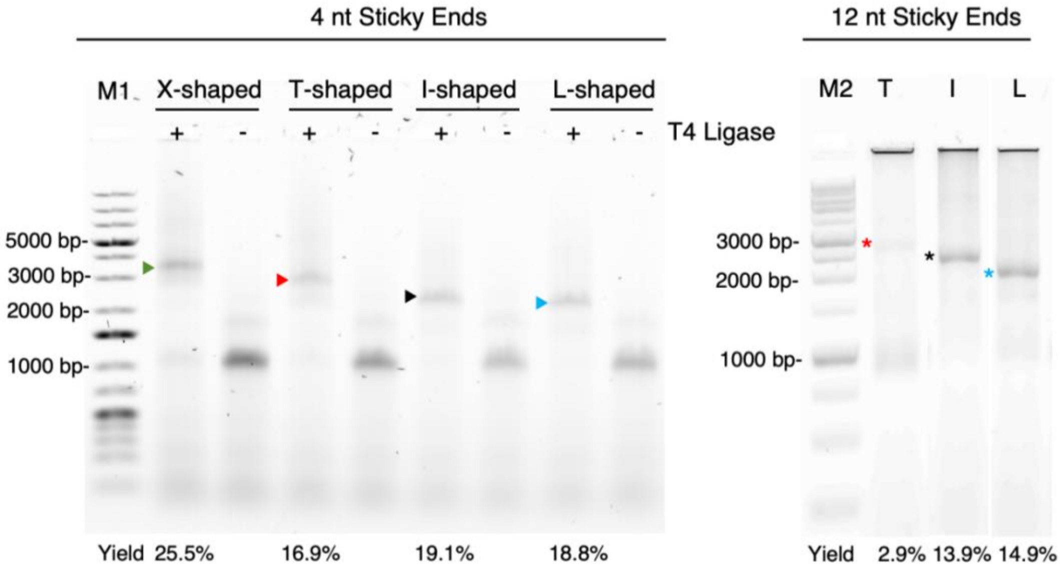

Figure S7. Gel electrophoresis results of the 5 × 5 lattices using ligation-induced method.

Left panel shows hierarchical assembly of 5 × 5 lattice units with T4 DNA ligase, forming higher-order structures as X-shaped pentamer (marked with green triangle), T-shaped tetramer (marked with red triangle), I-shaped trimer (marked with black triangle), and L-shaped trimer (marked with blue triangle), while controls without ligase show no reaction. "+" indicates samples treated with T4 DNA ligase, while "-" lanes serve as controls without ligase. Right panel shows a control group using elongated (12 nt) sticky ends. the yield of the T-shaped tetramer (T, marked with red asterisk) was measured at 2.9%, ~1/5 of the yield for the counterpart sample with shorter sticky ends, while the yield of I-shaped (I, marked with black asterisk) and L-shaped (L, marked with blue asterisk) trimer were also slightly lower than the structures with shorter sticky ends. Given the trend of a yield decline from I-shaped and L-shaped trimers to T-shaped tetramer using elongated sticky ends, the production of X-shaped pentamer were not investigated in this case. M1: 1 kb plus DNA Ladders; M2: 1 kb DNA Ladders.

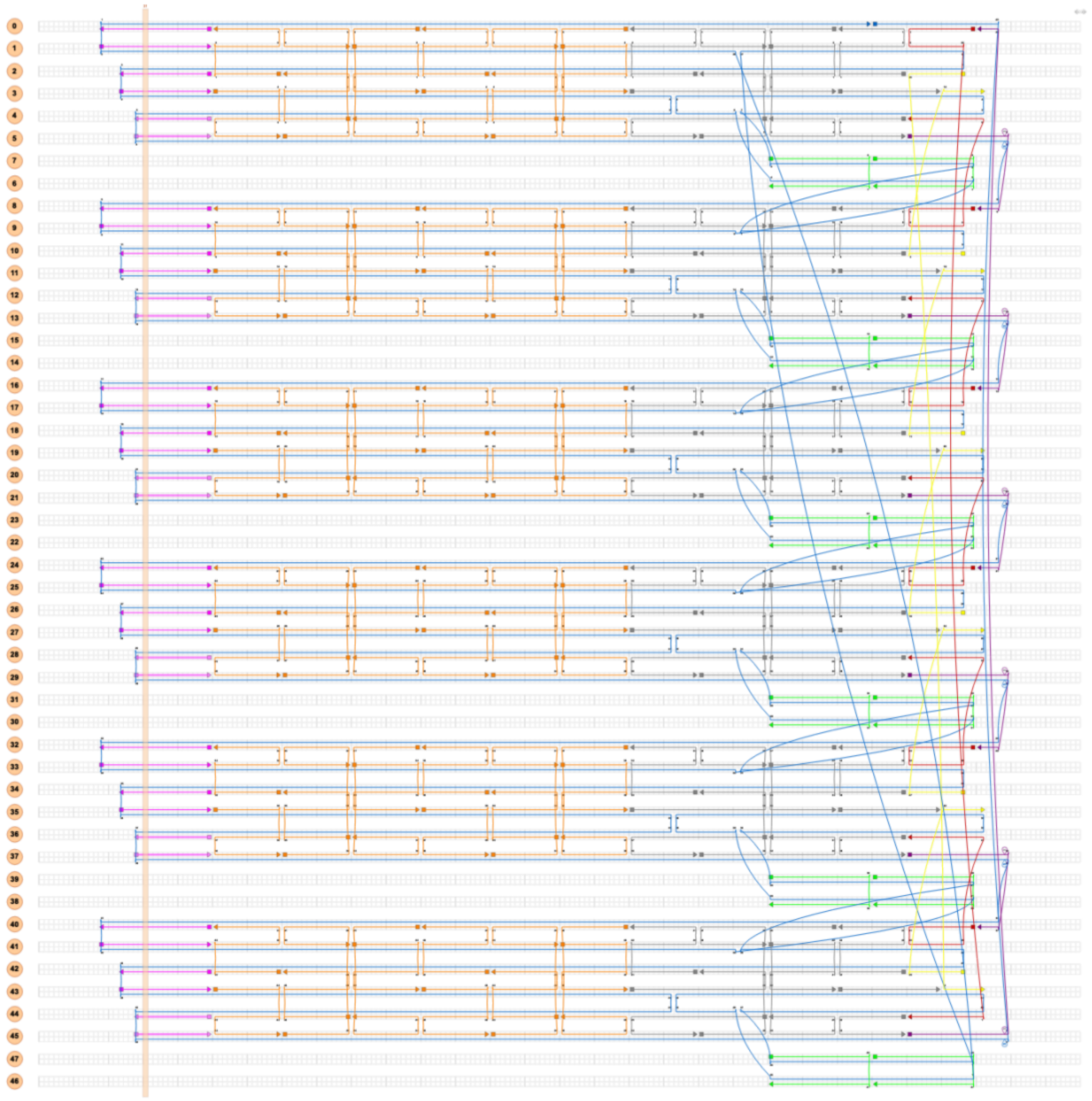

Figure S8. Cadnano diagram of snowflake origami unit.

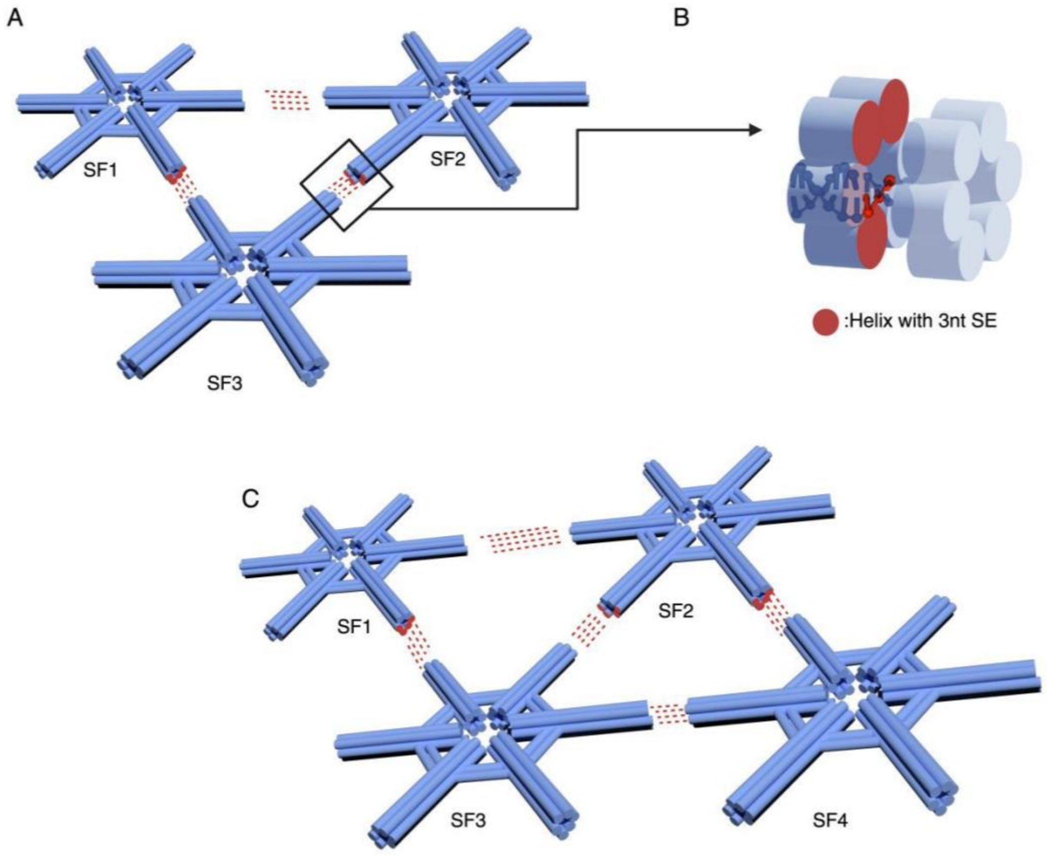

Figure S9. Design and assembly of origami units with 3-nt sticky ends.
(**A**) Illustration of snowflake (SF) origami units SF1, SF2, and SF3, showing two or three extending arms equipped with 3-nt sticky ends (four sticky ends per six-helix bundled arm as shown in red). (**B**) Close-up view of a single arm-to-arm sticky end cohesion scheme facilitating specific interactions between designated units. Sticky ends (shown in red) of three nucleotides were designed in four of the six helices for a certain arm. (**C**) Schematic showing the assembly pattern of origami units SF1, SF2, SF3, and SF4, demonstrating the formation of an organized multimer structure based on the designed sticky end cohesion.

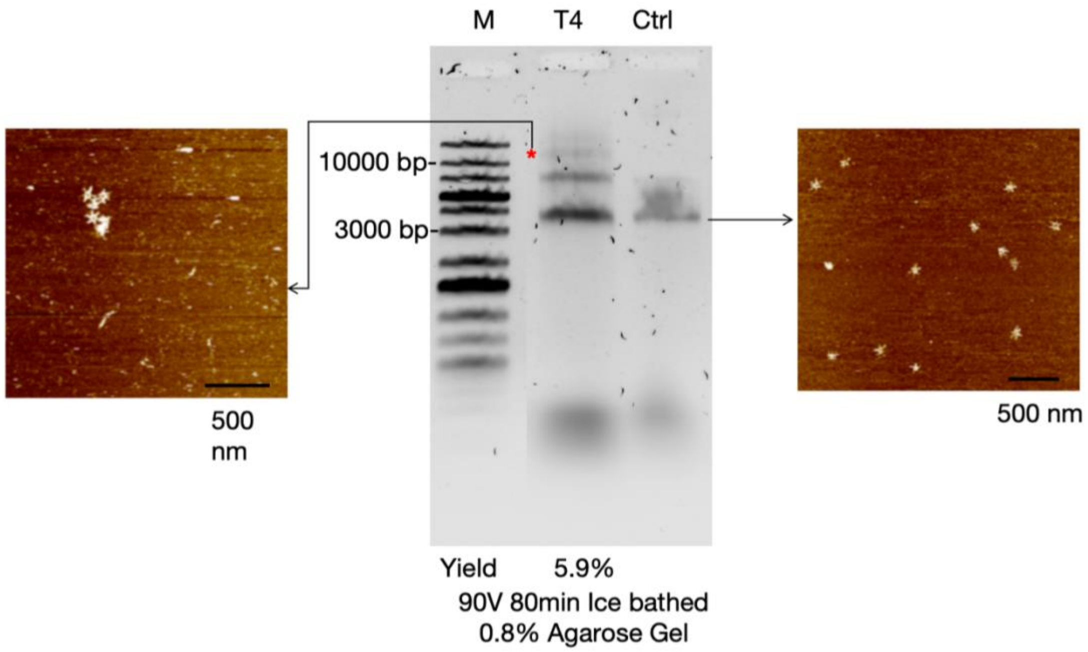

Figure S10. Gel electrophoresis and corresponding AFM images of ligation-induced assembly of snowflake trimer origami units.

The gel shows successful formation of the trimer structures (marked with red asterisk) from purified origami units in the presence of T4 DNA ligase (T4), while no assembly is observed for the control samples without ligase (Ctrl). DNA markers (M) are displayed on the left for reference. The results indicate that ligase treatment promotes the formation of higher-order structures, corroborating the TEM observations presented in Fig. 4G.

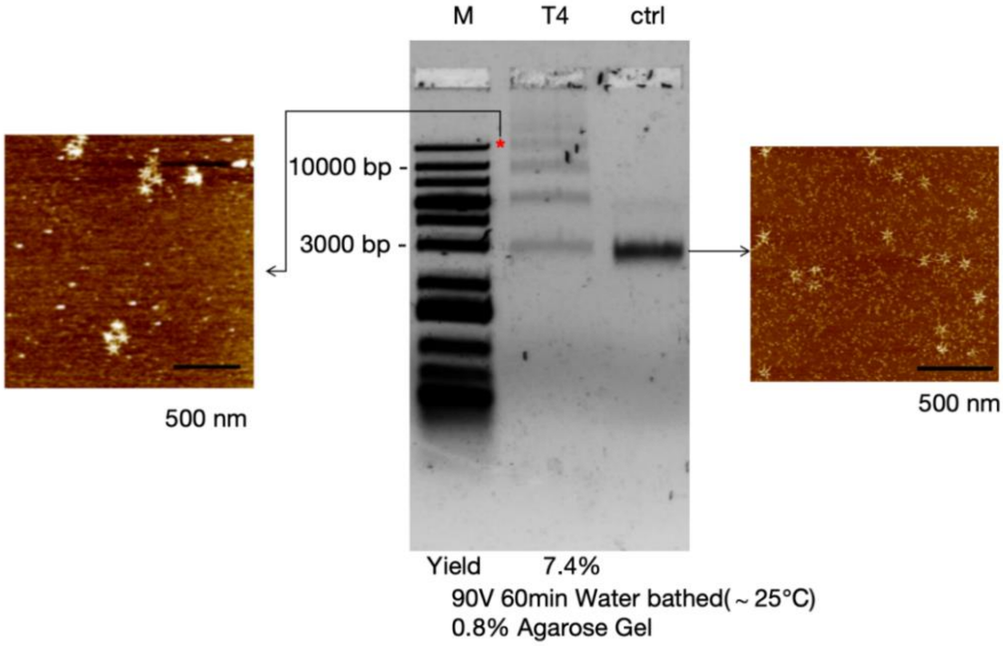

Figure S11. Gel electrophoresis and corresponding AFM images of ligation-induced assembly of snowflake tetramer origami units.

The gel shows successful formation of the tetramer structures (marked with red asterisk) from purifiedorigami units in the presence of T4 DNA ligase (T4), while no assembly is observed for the control samples without ligase (ctrl). DNA markers (M) are displayed on the left for reference. The results indicate that ligase treatment promotes the formation of higher-order structures, corroborating the TEM observations presented in Fig. 4H.

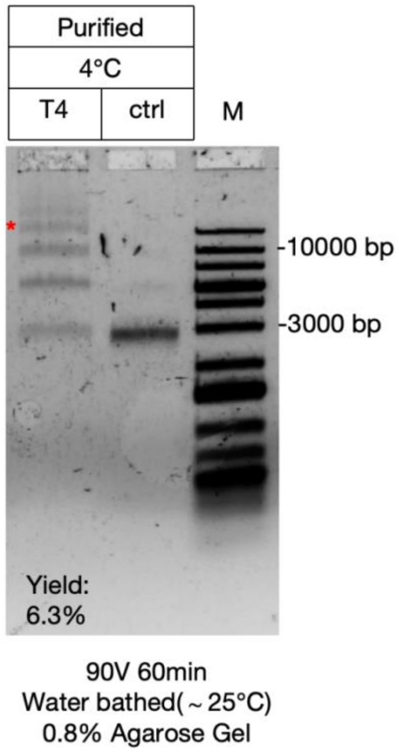

Figure S12. Gel electrophoresis of snowflake origami tetramer structures at lower temperatures.

Units were purified and incubated at 4 °C with (T4) or without (ctrl) T4 DNA ligase, as indicated. All other reaction parameters (e.g., buffer composition, reaction time) remained identical. M denotes the DNA marker (in base pairs). The red asterisks highlight the primary assembly products (~15000 bp), indicating successful formation of snowflake origami structures with T4 DNA ligase even at lower temperatures, while construct failed to self-assemble without ligation treatment.

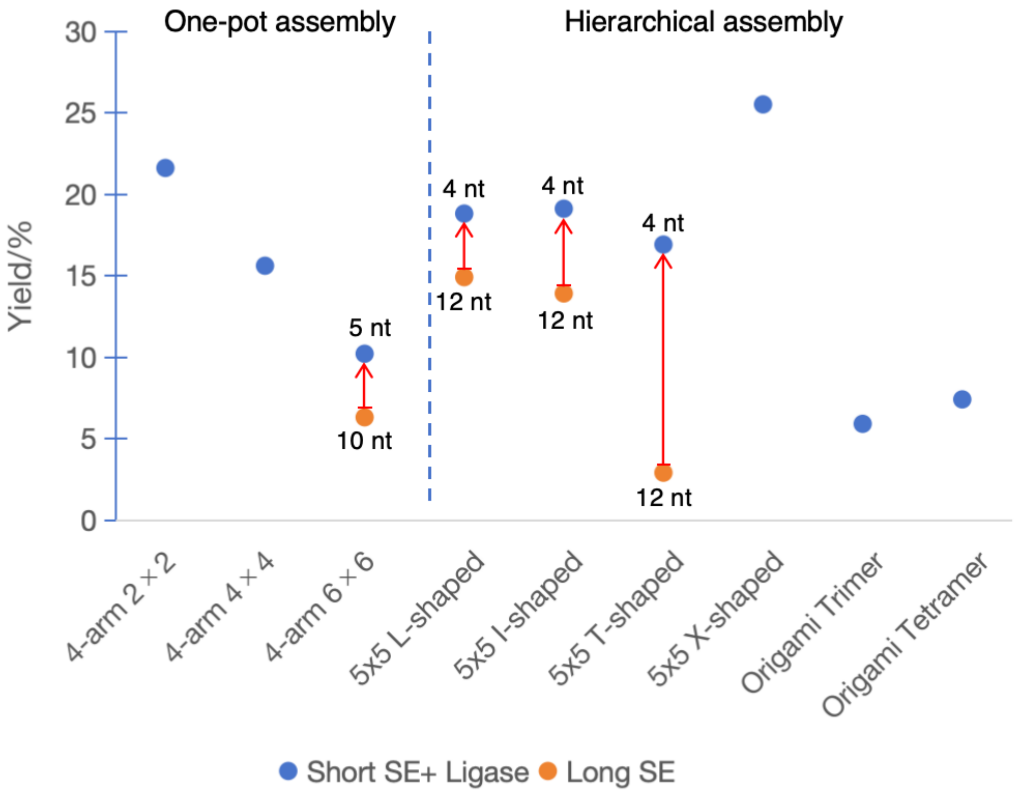

Figure S13. Assembly yields of design structures using ligation-induced method and traditional long sticky ends cohesion.

The assembly yields of various DNA nanostructures using two different approaches: ligation-induced assembly with short sticky ends (blue dots) and traditional assembly with long sticky ends (orange dots). Different DNA nanostructures were placed along the x-axis and divided into one-pot assembly group (left) and hierarchical assembly group (right), while the y-axis shows the assembly yield. Red arrows indicate the improvement in yield when using the ligation-induced assembly method compared to the traditional long sticky ends approach. The specific yield values for each condition are provided in Table S1. The results demonstrate that ligation-induced assembly with short sticky ends generally enhances the yield, particularly in hierarchical assembly processes.

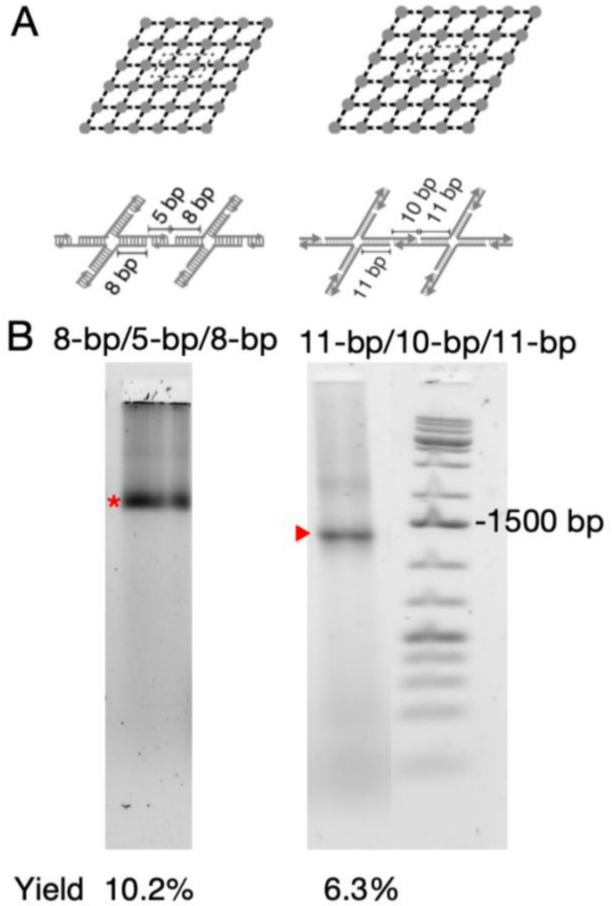

Figure S14. Comparison of 6×6 lattices formed via ligation-induced assembly and traditional long sticky-end cohesion.

(**A**) Schematic representation of 6×6 lattices assembled using two different strategies, ligation-induced assembly with short sticky ends (8-bp/5-bp/8-bp) (left) and traditional long sticky-end cohesion (11-bp/10-bp/11-bp) (right). (**B**) Corresponding native agarose gel electrophoresis results. The red asterisk points to the target 8-bp/5-bp/8-bp 6×6 lattice (yield: 10.2%), while the red triangle points to the target 11-bp/10-bp/11-bp 6×6 lattice (yield: 6.3%).

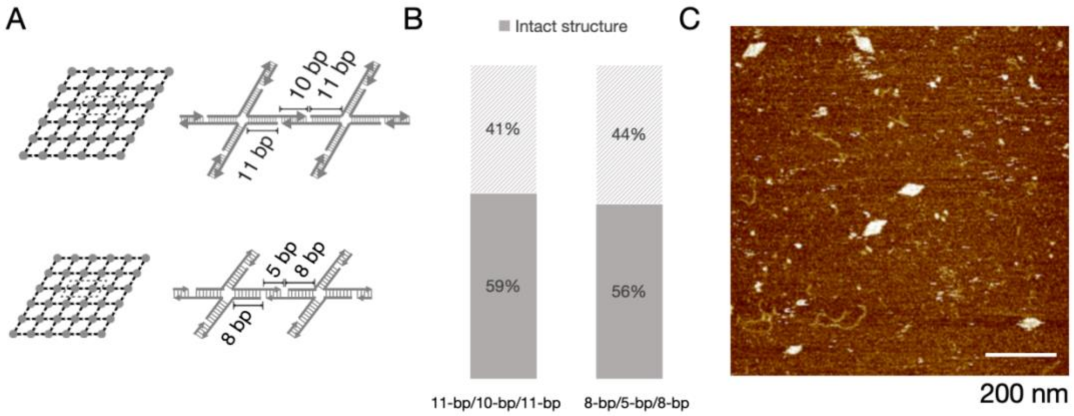

Figure S15. Comparison of structure integrity using ligation-induced method and traditional long sticky ends cohesion.
(**A**) Schematics of 6 × 6 lattice assembled from the traditional long sticky end cohesion method (11-bp/10-bp/11-bp, up) and the ligation-induced assembly strategy (8-bp/5-bp/8-bp, bottom). (**B**) The structural integrity is quantitatively assessed using AFM images, represented in the histogram. Statistics for 11-bp/10-bp/11-bp lattice (left bar, n=71) are derived from our previous work(2). Statistics for 8-bp/5-bp/8-bp lattice (right bar, n=52) are counted from AFM images, exampled as (**C**). (**C**) Full-size AFM images of 8-bp/5-bp/8-bp lattice from ligation-induced assembly. Scale bar:200 nm.

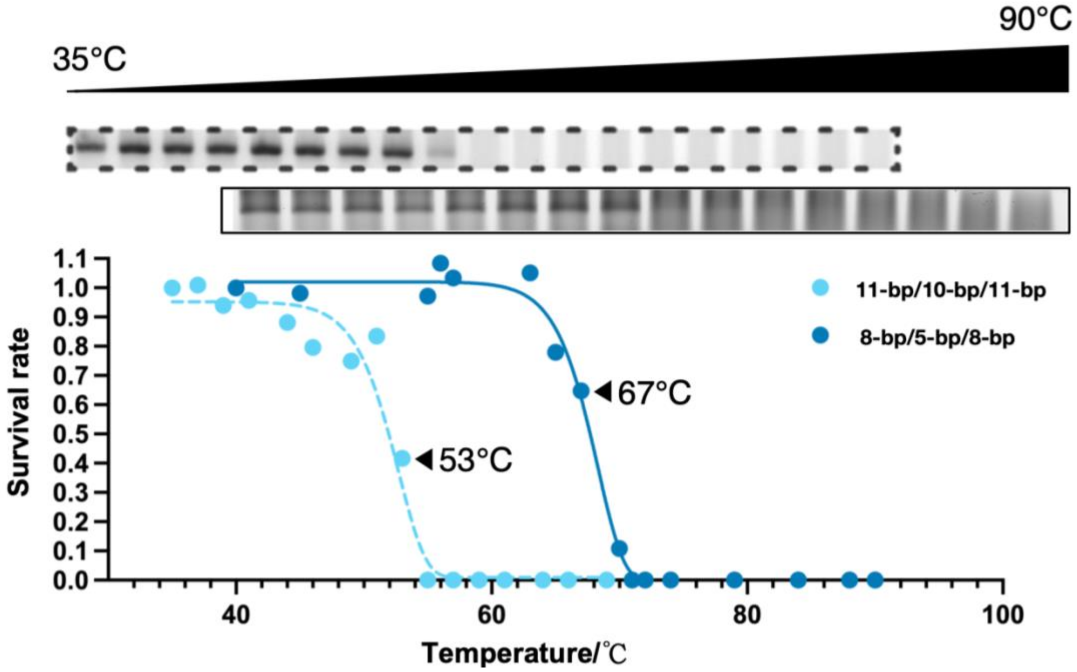

Figure S16. Thermal stability analysis of ligation-induced method and traditional long sticky ends cohesion.

Agarose gel results showing the stability of 6 × 6 lattices assembled from traditional long sticky ends cohesion method (11-bp/10-bp/11-bp, top panel)(2) and ligation-induced method (8-bp/5-bp/8-bp, bottom panel), corresponding to those described in S14. Structures were incubated across a temperature gradient from 35°C to 90°C. The 11-bp/10-bp/11-bp structure dissociated at lower temperatures, while the ligation-induced 8-bp/5-bp/8-bp lattice remains intact over a broader range. Melting curves derived from analysis of gel results. Dashed melting curve indicates the 11-bp/10-bp/11-bp lattice and solid melting curve indicate the ligated 8-bp/5-bp/8-bp lattice.

Supplementary Table

Table S1. Summary of design structures and assembly yields.

Structure	Nick pair Count	Sticky ends Lengths	Annealing Temperature	Yield
4-arm 2×2 Lattice	4	5 nt	30°C	21.6%
4-arm 4×4 Lattice	24	5 nt	30°C	15.6%
4-arm 6×6 Lattice	60	5 nt	30°C	10.2%
		10 nt	Ramp 60°C to 25°C	6.3%
5 × 5 lattice X-shaped pentamer	20	4 nt	30°C	25.5%
5 × 5 lattice T-shaped tetramer	15	4 nt	30°C	16.9%
		12 nt	37 °C	2.9%
5 × 5 lattice I-shaped trimer	10	4 nt	30°C	19.1%
		12 nt	37 °C	13.9%
5 × 5 lattice L-shaped trimer	10	4 nt	30°C	18.8%
		12 nt	37 °C	14.9%
Snowflake origami Trimer	12	3 nt	30°C	5.9%
Snowflake origami Tetramer	20	3 nt	30°C	7.4%

References:

1. Zadeh, J.N., Steenberg, C.D., Bois, J.S., Wolfe, B.R., Pierce, M.B., Khan, A.R., Dirks, R.M. and Pierce, N.A. (2011) NUPACK: Analysis and design of nucleic acid systems. *Journal of Computational Chemistry*, **32**, 170-173.
2. Bai, T., Zhang, J., Huang, K., Wang, W., Chen, B., Li, Y., Zhao, M., Zhang, S., Zhu, C., Liu, D. *et al.* (2022) Reconfiguration of DNA nanostructures induced by enzymatic ligation treatment. *Nucleic Acids Research*, **50**, 8392–8398.
